# A Therapeutic Challenge: Liddle's Syndrome Managed with Amiloride during Pregnancy

**DOI:** 10.1155/2014/156250

**Published:** 2014-08-25

**Authors:** Amelia Caretto, Liviana Primerano, Francesca Novara, Orsetta Zuffardi, Stefano Genovese, Maurizio Rondinelli

**Affiliations:** ^1^Department of Medicine, San Raffaele Scientific Institute and Università Vita-Salute, Via Olgettina 60, 20132 Milan, Italy; ^2^Department of Obstetrics and Gynecology, Hospital Buzzi, Via Castelvetro 32, 20154 Milan, Italy; ^3^Department of Molecular Medicine, University of Pavia, Via Forlanini 14, 27100 Pavia, Italy; ^4^Diabetes and Endocrine Unit, Cardiovascular and Metabolic Department, IRCCS Multimedica, Via Milanese 300, 20099 Sesto San Giovanni, Italy

## Abstract

Liddle's syndrome (LS) is a rare heritable form of hypertension that often affects young patients. It is caused by gain-of-function mutations of the kidney epithelial sodium channel (ENaC) and it is classically associated with hypokalemia and suppression of renin and aldosterone. LS is characterized by responsiveness to ENaC inhibitors but not to mineralocorticoid receptor inhibitors. Consequently the most effective treatment is amiloride. This drug is not used in pregnancy, as it has not been sufficiently studied during gestation. However for pregnant LS patient amiloride is the most effective drug in decreasing blood pressure. Herein we report the case of a LS patient, who has been followed up by a multidisciplinary teamwork during her first pregnancy. Hypertension worsened after the 25th week of gestation and amiloride was safely administered, firstly in combination with hydrochlorothiazide (the only formulation commercially available in Italy) and, thereafter, as a single drug. Genetic testing was performed in the patient's family in order to support diagnosis and clinical management.

## 1. Introduction 

Liddle's syndrome (LS) is a rare autosomal-dominant form of salt-sensitive hypertension due to activating mutations in the epithelial sodium channel (ENaC) of the distal nephron. Characteristic features include low levels of plasma renin activity and aldosterone, hypokalemic alkalosis, and responsiveness to ENaC inhibitors but not to mineralocorticoid receptor inhibitors [1].

The ENaC complex is composed of three subunits (*α*, *β*, *γ*) each encoded by a specific gene (*SCNN1A, SCNN1B, SCNN1C*) and consisting of two transmembrane regions, a large extracellular domain and cytoplasmic amino and carboxyl termini [2]. The majority of causative mutations alter or delete a proline rich segment (PY motif) in the carboxyl cytoplasmic tail of *β* or *γ* subunit, responsible for negative regulation of the channel, therefore resulting in its overactivation [3].

Here, we report the case of a young woman clinically diagnosed with LS who came to our attention just before pregnancy. Multidisciplinary teamwork allowed tailored and effective control of hypertension during pregnancy.

## 2. Case Report

### 2.1. Clinical History

A 24-year-old woman was referred to our hospital for evaluation in June 2012 following an episode of hypertensive crisis (blood pressure (BP) 188/125 mmHg). She was diagnosed with LS in 1999 after the discovery of hypokalemia, but genetic test to confirm diagnosis had never been performed. For hypertension, she was treated with lacidipine 6 mg daily that brought about reduction but not normalization of BP. Despite the fact that amiloride is the drug of choice for LS treatment [2], in Italy it is commercially available only in fixed formulation with hydrochlorothiazide; therefore a calcium channel blocker was preferred in this patient.

Early after the first evaluation, the patient became unintentionally pregnant. Therapy with lacidipine allowed BP control until the 25th gestational week, when the patient was hospitalized for a hypertensive crisis (BP 160/110 mmHg associated to a strong headache). The absence of proteinuria ruled out preeclampsia, as possible differential diagnosis. The patient started extended release (ER) nifedipine 20 mg twice a day plus a fix dose combination of amiloride 2.5 mg/hydrochlorothiazide 25 mg once a day, doubling gradually the dosage, achieving BP normalization in the absence of any adverse effect on pregnancy.

At 37th week of pregnancy ultrasound scan examination revealed fetal growth retardation (as testified by an abdominal circumference at the 15th percentile, according to customized growth chart) together with anomalies in the umbilical artery Doppler velocimetry. Upon a second hypertensive crisis (BP 150/100 mmHg), the patient was again hospitalized and started amiloride 7.5 mg once a day, as we were finally able to obtain the oral formulation with amiloride alone. Because of symptoms (headache and visual scotoma) persistence, the dosage of amiloride was increased to 15 mg daily achieving an optimal control of BP.

On February 28 2013 a planned labor induction was started. Because of BP increase (170/120 mmHg) during labor, despite the use of nifedipine plus amiloride, cesarean section delivery was performed. The newborn was a healthy male child (weight: 2260 grams, APGAR score: 9-10, blood pH: 7.28), and, by third day after delivery, patient's BP was normalized (125/85 mmHg).

### 2.2. Gene Analysis and Study of the Family

**Figure 1:**
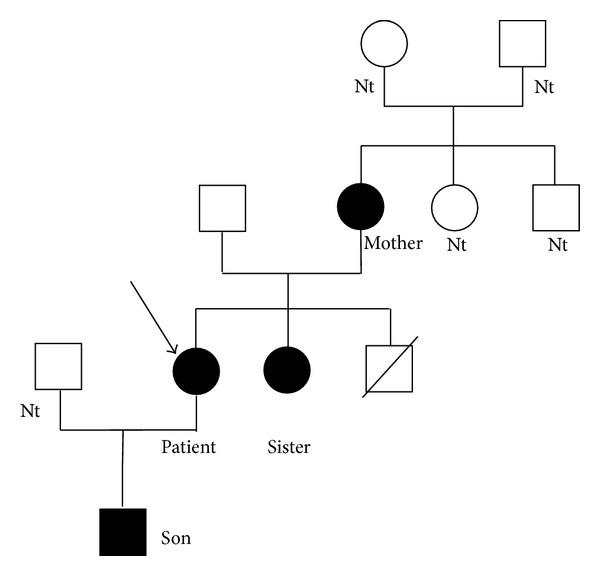
Pedigree of the proband's family. Squares indicate males and circles females. Individuals with* SCNN1B* mutation are shown as filled figures. The proband is indicated by the arrow. Nt: not tested.

The proband's maternal line, of Sicilian origin, was highly suspected to be affected by LS, since the grandmother died at age 47 of cerebral hemorrhage and the mother suffered from arterial hypertension and had an ischemic stroke at age 38. Proband's sister showed no symptoms of high BP or hypokalemia. The proband, her son, mother, and sister underwent genetic analysis and the resulting pedigree is shown in Figure 1.

The analysis showed a heterozygous C to T mutation at codon 617 in exon 13 of* SCNN1B* gene in all subjects analyzed. This mutation caused proline to leucine substitution in the PY motif of the *β* subunit (P617L), causing an overactivation of ENaC as recently described by Rossi et al. [4].

## 3. Discussion

Pregnancy is a critical condition for a woman affected by LS, as BP can worsen during gestation leading to adverse maternal and neonatal outcomes. Preexisting hypertension is in fact a risk factor for preeclampsia [5], even if a causal relationship between LS chronic hypertension and preeclampsia has never been confirmed. Indeed, our patient did not develop preeclampsia, as she never developed proteinuria but BP worsened during gestation, especially after the 25th gestational week. Such a situation caused a therapeutic challenge: few antihypertensive drugs are safely administered in pregnancy and amiloride has no indication in this clinical condition. Usually the preferred agents for first-line treatment of hypertension during pregnancy are methyldopa or calcium channel blockers [6], but for LS hypertension amiloride represents the best therapeutic choice, because of its direct action on renal ENaC and its potassium-sparing effect. There are no randomized clinical trials exploring the safety profile of amiloride during gestation, and it is known that it crosses placenta in modest amounts. However, the Food and Drug Administration (FDA) classifies amiloride in the category B of teratogenicity, and studies in rats showed only limited toxicity at doses much higher than those used in humans [7]. Based on these data, we decided to prescribe amiloride to our patient, with a maximum dosage of 15 mg daily, starting from the 25th gestational week, when BP was not anymore controlled by calcium channel blocker. Importantly, we did not register maternal or fetal adverse events related to the drug. We observed only a mild decrease in intrauterine growth, but this was most likely to be the effect of hypertension, rather than of amiloride administration. Our observation is in concordance with those reported in other published case reports of women treated with amiloride during pregnancy without complications. Disease backgrounds were different from LS, being Bartter's syndrome [8, 9] and Gitelman's syndrome [10] and the maximum dose of amiloride used was 30 mg daily, which is higher than that we used in our patient [8, 9]. Amiloride was effective and well tolerated also in a pregnant woman with aldosteronism secondary to bilateral adrenal hyperplasia [11]. In this latter report, the patient had a difficult first pregnancy, treated with several lines of antihypertensive drugs (including methyldopa, labetalol, and nifedipina) which were, however, unable to control BP, leading to fetal growth retardation and ultimately to miscarriage. In sharp contrast, treatment with amiloride 10–15 mg daily allowed reaching an optimal BP control, and consequently fetal health, during the following two pregnancies. These observations reinforce the safety profile of amiloride during pregnancy and support our hypothesis that the decrease in intrauterine growth observed in our patient's child was likely due to uncontrolled hypertension or other factors, rather than to amiloride. These results are further corroborated by the observations of Al-Ali et al. [12], who described a pregnancy complicated by primary aldosteronism and adrenal adenoma in which amiloride proved to be a safe alternative to the mineralocorticoid antagonist spironolactone that is contraindicated in pregnancy.

In our case, amiloride was at first used in fixed combination with hydrochlorothiazide, since the single drug formulation is not commercially available in our country. As for amiloride, hydrochlorothiazide is in FDA category B of teratogenicity [7]. Diuretics in pregnancy may cause electrolyte imbalance and volume depletion with consequent uteroplacental hypoperfusion. In our patient, however, electrolyte values were tightly controlled and supplemented when needed, while volume depletion was balanced by pregnancy-induced plasma volume expansion and by LS-specific chronic volume expansion caused by constitutive activation of ENaC. Thus, in the setting of LS, also hydrochlorothiazide proved to be safe and manageable during pregnancy.

Physiological pregnancy sodium retention mediates plasma volume increase necessary for fetus development. It has been demonstrated in rats that the main mechanism of sodium retention is the increase of the number of *α* ENaC subunits and that the enhanced ENaC activity, especially marked in the later stages of normal pregnancy, is mineralocorticoid-dependent [13]. These findings might explain why we observed a progressive deterioration of BP control during our patient's pregnancy: despite the fact that ENaC is constantly activated in LS, pregnancy hormonal changes may provide additional stimuli to increase the number of channels, especially in the latest part of gestation, so that the therapeutic dose of amiloride might be different according to the gestational period.

Our proband's mutation *β*P617L was firstly described by Rossi et al. [4] in a 19-year-old man and in his family of Sicilian-Calabrian origin (Southern Italy) and then confirmed by the same authors [14] in another independent Sicilian family affected by LS. Our proband's kindred have also Sicilian origin, as the other families are known as carriers of the *β*P617L mutation. The patient is unaware of any biological relationship of her kindred with the other carriers, even if it cannot be definitively ruled out. Further investigations could be useful to corroborate the hypothesis of a “founder effect.” As far as we know from the literature, no other disease mutation has been reported to be specific of the Italian population.

We confirm the known phenotypic variability of LS, observed also in patients of the same kindred [14], while our patient and her mother showed early onset hypertension requiring intensive pharmacological therapy, and her sister did not show alterations in BP values. Many factors could account for this variability, including ENaC allele-specific effects, allelic variants of other genes interfering with BP and potassium control, lifestyle, and dietary habits.

Although LS often affects women of childbearing age, there are few reports dealing with therapeutic management of LS during pregnancy [15]. In our hands, amiloride proved to be safe and effective when no other antihypertensive drugs were effective. Our experience highlights also the importance of making a precise clinical and genetic diagnosis of LS in order to manage patients with a multidisciplinary team.
